# Enhancement of pattern quality in maskless plasmonic lithography via spatial loss modulation

**DOI:** 10.1038/s41378-023-00512-4

**Published:** 2023-03-30

**Authors:** Dandan Han, Sen Deng, Tianchun Ye, Yayi Wei

**Affiliations:** 1 University of Chinese Academy of Sciences, School of Integrated Circuits, Beijing, 100049 China; 2 Chinese Academy of Sciences, Institute of Microelectronics, Beijing, 100029 China

**Keywords:** Nanophotonics and plasmonics, Nanophotonics and plasmonics

## Abstract

Plasmonic lithography, which uses the evanescent electromagnetic (EM) fields to generate image beyond the diffraction limit, has been successfully demonstrated as an alternative lithographic technology for creating sub-10 nm patterns. However, the obtained photoresist pattern contour in general exhibits a very poor fidelity due to the near-field optical proximity effect (OPE), which is far below the minimum requirement for nanofabrication. Understanding the near-field OPE formation mechanism is important to minimize its impact on nanodevice fabrication and improve its lithographic performance. In this work, a point-spread function (PSF) generated by a plasmonic bowtie-shaped nanoaperture (BNA) is employed to quantify the photon-beam deposited energy in the near-field patterning process. The achievable resolution of plasmonic lithography has successfully been enhanced to approximately 4 nm with numerical simulations. A field enhancement factor (*F*) as a function of gap size is defined to quantitatively evaluate the strong near-field enhancement effect excited by a plasmonic BNA, which also reveals that the high enhancement of the evanescent field is due to the strong resonant coupling between the plasmonic waveguide and the surface plasmon waves (SPWs). However, based on an investigation of the physical origin of the near-field OPE, and the theoretical calculations and simulation results indicate that the evanescent-field-induced rapid loss of high-*k* information is one of the main optical contributors to the near-field OPE. Furthermore, an analytic formula is introduced to quantitatively analyze the effect of the rapidly decaying feature of the evanescent field on the final exposure pattern profile. Notably, a fast and effective optimization method based on the compensation principle of the exposure dose is proposed to reduce the pattern distortion by modulating the exposure map with dose leveling. The proposed pattern quality enhancement method can open new possibilities in the manufacture of nanostructures with ultrahigh pattern quality via plasmonic lithography, which would find potentially promising applications in high density optical storage, biosensors, and plasmonic nanofocusing.

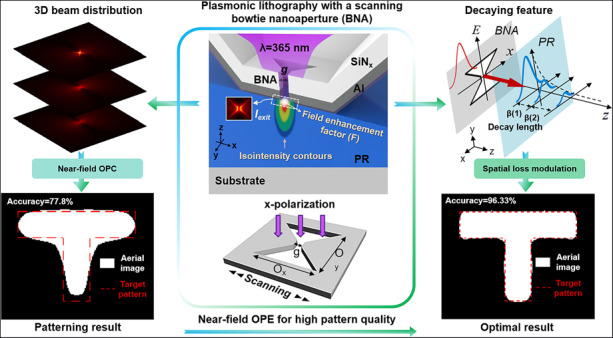

## Introduction

Optical lithography technology plays an indispensable role in the semiconductor industry, especially for the very-large-scale-integration (VLSI) manufacturing^1–4^. With the scaling of integrated circuits in pace with Moore’s law, the imaging quality of optical lithography has been adversely affected by the proximity effects, particularly when the critical dimension (CD) reaches the achievable resolution of optical lithography^5^. Proximity effects refer to the variations in the target feature as a function of the proximity of other nearby features. Previous studies have found that the physical origins of the proximity effects in optical lithography are attributable to the low-pass filtering properties of the lenses used, which cut off the high spatial-frequency (*k*) information and lead to serious image distortions, i.e., corner rounding, line-width variation, and line-length shrinking^6^. Consequently, to reduce the influence of the proximity effect on image quality and enhance the resolution and image contrast of the generated aerial images in optical lithography, many types of resolution enhancement techniques (RETs) have been proposed, such as off-axis illumination (OAI), phase-shifting masks (PSMs), optical proximity correction (OPC), antireflective coating (ARC), double exposure methods, and inverse lithography^7–14^. Essentially, these RETs aim to reduce the value of *k*_*1*_, which represents the ability to approach physical resolution limits depending on the lenses, photoresist (PR) materials, equipment, and process controls in the manufacturing. Meanwhile, the proximity effects caused by the electron scattering can also degrade the pattern quality of electron-beam lithography (EBL)^15,16^. Different proximity effect correction (PEC) methods based on the estimation of the point-spread function (PSF) have been adopted to overcome the proximity effects and achieve high-quality patterns of EBL^17^.

Plasmonic lithography is a cost-effective and promising technique in manufacturing subdiffraction-limited nanopatterns for broad applications^18–29^. However, as the CD is reduced to well below 100 nm and the pattern shapes become more complex and diverse, the near-field optical proximity effect (OPE) will eventually become a limiting factor of pattern quality in the plasmonic lithography since the near-field OPE becomes more serious and it further aggravates the pattern image distortion. Therefore, it is essential to minimize the near-field OPE in order to achieve the highest pattern resolution and fidelity possible by the plasmonic lithographic process. In mask-based plasmonic lithography, OAI, PSMs, and OPC methods have been developed to relieve the image distortion problems, and in contrast, a simple rule-based OPC method has been adopted to improve the sharpness of corners and edges, and line shortening in maskless plasmonic lithography^30–34^. However, a detailed understanding of the near-field OPE in plasmonic lithography, for accurate nanometer-scale patterning with quantitative proximity correction and compensation for image distortion remains an open challenge.

Attributing to its extraordinary ability to generate strong light confinement and dramatic field enhancement in a nanometer scale, a metallic bowtie-shaped nanoaperture (BNA) consisting of two open arms separated by nanoridges (form a metal-insulator-metal (MIM; metal-air-metal) structure) has been utilized for nanolithography, plasmonic nanofocusing, and high-density data storage^35–39^. For maskless plasmonic lithography with a BNA, the BNA serves as a local near-field optical source, and at the exit of the BNA, surface plasmon waves (SPWs) are exploited to generate high spatial-frequency (*k*) evanescent fields in the photoresist (PR) layer^25,28,40^. The three-dimensional field distribution of a BNA has been characterized using quasi-spherical waves (QSWs) and surface plasmon polaritons (SPPs), and the SPWs are determined by the evanescent mode of the QSWs and SPPs^41^. However, the SPWs are surface-confined evanescent waves. Despite the SPWs having the ability to carry high spatial frequency optical information to the image plane, the rapid divergence of the light scattered from the plasmonic BNA results in the amplitude of the SPWs decaying exponentially with exposure distance away from the surface of the PR layer, which will inevitably cause that the depth of the image plane of plasmonic lithography is limited in the range of a few nanometers to several tens of nanometers. Specifically, the rapidly decaying feature of SPWs is bound to cause the loss of high-*k* information in the PR layer, which implies that the loss of high-*k* information will lead to an irrecoverable loss of resolution and pattern fidelity with the exposure distance. Therefore, to analyze the quality of the near-field patterning with arbitrary shape, it is necessary to investigate the underlying physics of the evanescent-field-induced high-*k* information loss.

In this paper, we studied the physics behind the near-field diffraction limit from an evanescent-field-based patterning system, with the goal of finding the ultimate achievable pattern fidelity. Numerical calculations have been carried out to evaluate the achievable resolution of the plasmonic lithography and quantitatively analyze the near-field enhancement effect and the size dependence of the plasmonic near-field, with the aim of estimating the PSF with accuracy and efficiency, because the PSF consists of the spatial distribution of the exposure dose density deposited in the PR layer and contains all physical and chemical phenomena that contribute to the near-field OPE during the exposure process. An analytical formula was proposed to quantitatively analyze the effect of the rapidly decaying feature of the evanescent field on the near-field OPE and the theoretical limit of the pattern fidelity. In view of the features of the near-field OPE in plasmonic lithography, a fast effective method for correcting the evanescent-field-induced rapid loss of high-*k* information through exposure dose compensation in advance with exposure dose map is put forward, and its effectiveness has been verified by simulation results. It is expected that the proposed optimization method can provide useful guidance in minimizing pattern errors and improving the practical application of plasmonic lithography.

## Modeling

### Optical resolution limit in plasmonic lithography

Unlike conventional optical lithography, the pattern profile generated by the near-field of a plasmonic BNA is mainly determined by the enhanced evanescent field with rapidly decaying feature penetrating into the PR layer. Moreover, according to the near-field lithography modeling, the exposure profile of PR is identical to that of the exposure dose distribution. Therefore, the achievable resolution can then be defined by the full width at half maximum (FWHM) of its resulting spatial distribution of exposure dose deposition, i.e., the PSF^22,28,34^. When the plasmonic lithography system produces nanoscale target patterns with an arbitrary shape, the corresponding exposure dose distribution is computed by the convolution between the target pattern and the PSF, which is radially symmetric and displays how the beam energy is distributed throughout the PR when a single point is exposed. Therefore, knowledge of the PSF is useful for pattern profile prediction in the patterning process^29^. Significantly, estimation of the PSF with high accuracy and efficiency is crucial in the quantitative analysis of the achievable optical resolution and the near-field OPE in plasmonic lithography.

**Fig. 1:**
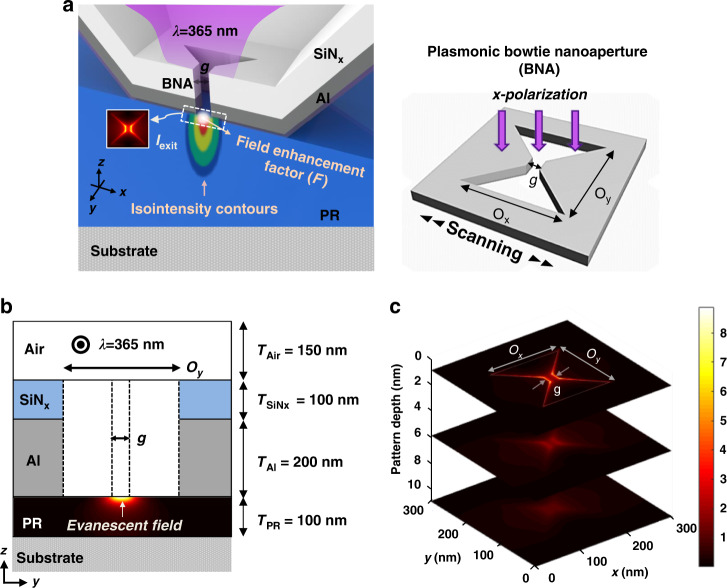
Plasmonic lithography with a scanning contact probe and the field distribution in the PR layer. **a** Schematic of the maskless plasmonic lithography system with a scanning plasmonic BNA. The optical source, which is a linearly *x*-polarized laser with 365 nm wavelength, is incident on a plasmonic BNA. The plasmonic BNA is perforated at the tip apex of a scanning probe. The outline dimension is *O*_*x*_ *=* *O*_*y*_ *=* 150 nm with various ridge-gap sizes (*g*) from 4 to 24 nm. **b** Schematic configuration of the simulated plasmonic lithography with a BNA. The transmitted light in the PR layer is an evanescent field, and a nanocandle-like extreme photon density profile can be observed. **c** Three-dimensional field intensity distribution in the PR layer. The small gap between the nanoridges of the plasmonic BNA acts as an electromagnetically ultra-intense hot-spots, and the open arms allow the electromagnetic (EM) fields to circulate

To understand the limitations of the approaches reported in previous studies, we consider a well-known plasmonic lithography scheme, and a schematic configuration of the plasmonic lithography with a BNA is exhibited in Fig. 1a. As illustrated in Fig. 1a, b, the plasmonic BNA is embedded into aluminum (Al,$\varepsilon _{Al} = - 19.4 + 3.6i$) and silicon nitride (SiNx, $\varepsilon _{SiNx} = 2.1$) by using the focused-ion-beam (FIB) milling method, and its geometric parameters are defined by the outline dimensions *O*_*x*_ and *O*_*y*_, and the ridge gap size *g*. A finite differential time domain (FDTD, Ansys Lumerical v2021) calculation is performed to characterize the PSF, that is, a three-dimensional intensity field distribution generated by the plasmonic BNA at a wavelength of 365 nm with a transverse magnetic (TM) polarized incident beam on the PR layer ($\varepsilon _{PR} = 2.9$). As shown in Fig. 1c, the generated near-field PSF has a complex beam distribution identified by both evanescent field and propagating wave components emerging from the plasmonic BNA, and different decaying characteristics in the lateral and vertical directions coexist within the near-field region. These results imply that the optical resonance of the plasmonic BNA is strongly dependent on its geometry, and the complicated and asymmetrical field decaying features of the PSF may be the most significant contributors to the near-field OPE in plasmonic lithography.

It has been demonstrated that there are two types of optical resonances that dominate in the transmission spectrum of a plasmonic BNA: Fabry-Pérot-like resonance (labeled ‘F-P’) and plasmonic resonance (labeled ‘Plasmonic’)^28,42^. The F-P-like resonance, excited in the ultraviolet regime, is dependent on the thickness of the Al film. The electric field distribution at the F-P-like resonance shows a standing wave characteristic, which is similar to that of a Fabry-Pérot cavity; thus, the F-P-like resonance wavelength is generally thought to be determined by the Fabry-Pérot cavity condition $\lambda = 2{{{\mathrm{n}}}}_{{{{\mathrm{eff}}}}} \cdot {{{\mathrm{t}}}}/{{{\mathrm{m}}}}$, where *n*_*eff*_ is the effective refractive index, *t* is the metal film thickness, and *m* is an integer. However, the plasmonic resonance, appears in the visible and near-infrared spectral regimes, which is closely related to the gap size (*g*) of the plasmonic BNA and it has a representative field distribution of the fundamental plasmonic resonance of a BNA.

**Fig. 2:**
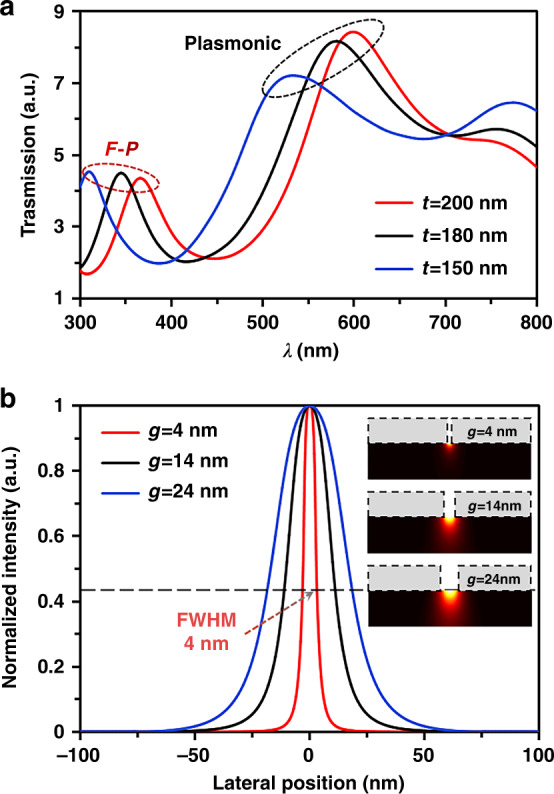
The achievable optical resolution of the maskless plasmonic lithography. **a** Transmission efficiency of the plasmonic BNA dependence on the incidence wavelength (*λ*) under different Al thicknesses. The geometry of the plasmonic BNA is given by *O*_*x*_ *=* *O*_*y*_ *=* 150 nm, and *g* = 20 nm. **b** Normalized intensity profiles along the *y*-direction at the PR surface with nanoridge gap sizes of 4 nm, 14 nm, and 24 nm, indicating an ultimate resolution of 4 nm

The transmission spectral resonance of a plasmonic BNA is studied numerically with different Al film thicknesses, as shown in Fig. 2a. The F-P-like resonance and plasmonic resonance is observed in the ultraviolet and visible spectral regime, respectively. Because the F-P-like resonance appears at a shorter wavelength than the plasmonic resonance; therefore, under 365 nm illumination, the F-P-like resonance plays a dominant role in enhancing the transmission efficiency of the plasmonic BNA. It is noted that the F-P-like resonance is red-shifted from 300 nm to 400 nm, as the thickness of the Al film increases from 150 nm to 200 nm. Evidently, the F-P-like resonance peak is 365 nm when the Al thickness is set as 200 nm, which is in good agreement with the Fabry-Pérot cavity condition.

The achievable optical resolution of plasmonic lithography can be given by the FWHM of the PSF used for patterning, as it represents the capability of high-density recording^22,28,34^. Moreover, since the gap size of the plasmonic BNA plays a very significant role in light confinement and field enhancement and determines the spatial size of the generated near-field beam spot, we numerically study the gap size dependence of the PSF. The calculated PSF curves for several plasmonic BNAs with ridge gaps of 4, 14, and 24 nm are plotted in Fig. 2b. Considering the asymmetrical field distribution determined by the plasmonic BNA and the evanescent field decays rapidly in the normal direction, we evaluated the achievable resolution along the *y*-direction at the Al-PR interface (*z* *=* 0 nm). Notably, the achievable optical resolution limit is ~4 nm for the plasmonic BNA, which is approximately equal to the physical dimension of the gap size. Meanwhile, thanks to advanced nanofabrication techniques such as the EBL and FIB milling, the ridge gap size of a plasmonic BNA has been successfully reduced to 4 nm^43^. As a consequence, we can conclude that plasmonic lithography has great potential to generate nanopatterns with ultrahigh-resolution and arbitrary shapes. Even more importantly, because the optical resolution varies greatly with change in the gap size, quantitative analysis of the size dependence of resolution limit, or the size tunability of the near-field is very important for understanding the root causes of near-field OPE and thereby enhancing the pattern quality in plasmonic lithography.

### Size dependence of near-field enhancement with a plasmonic BNA

Breaking the diffraction limit is always an appealing topic in optical lithography due to the urge for a higher subwavelength resolution. As an effective solution, the nanoridge apertures based on the plasmonic effect, also known as surface plasmon polaritons (SPPs), can resonantly amplify the evanescent waves, achieve strong field enhancement, and obtain ultrahigh resolution. Recently, a plasmonic BNA has been demonstrated that shows the nanoscale confinement of light is due to the strong coupling effect between the highly localized SPPs and the plasmonic waveguide mode^41^. When the incident light is polarized across the ridge gap of BNA, the photon energy is coupled into the gap region and thus a nanoscale beam spot is generated. There has been extensive work indicating that the achievable resolution of the generated beam spot is also limited by the fabrication capability of producing a small gap in the BNA, and on the other hand, the enhancement of the electric field shows a strong dependence on the gap size. Surprisingly, the size tunability of the near-field enhancement for a plasmonic BNA has not been characterized.

**Fig. 3:**
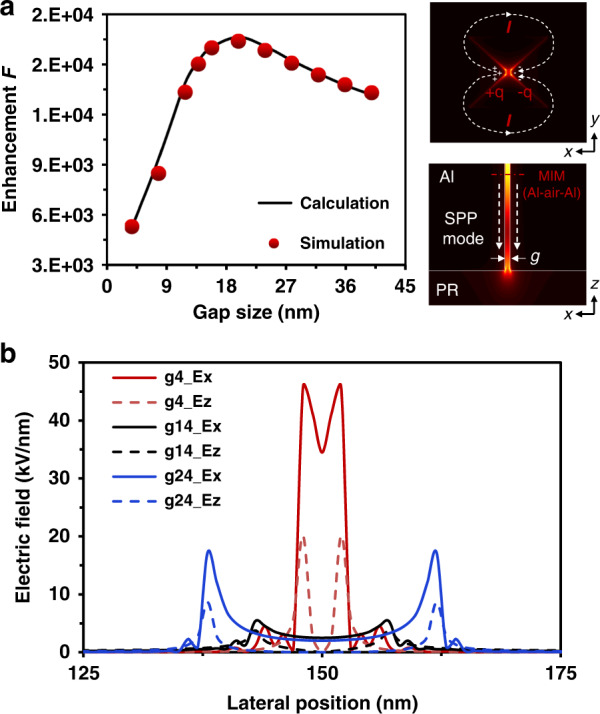
Quantitative analysis of the near-field enhancement effect in plasmonic lithography. **a** Near-field enhancement at the exit of a plasmonic BNA. The enhancement factor F is plotted as a function of the gap size, and calculation data (black solid line) and simulation results (red circles) are shown. A volcano trend can be seen with a maximum of ~20 nm. The simulated electric field *|E*_*xy*_*|* (background color), surface current (dotted line), and charges (signs) at the exit of a plasmonic BNA are shown in the top right, and the cross-sectional electric field *|E*_*xz*_*|* is shown in the lower right. **b** Calculated transmission amplitudes of the electric field components, i.e., *|E*_*x*_*|* and *|E*_*z*_*|*, at the exit of a plasmonic BNA with different nanoridge gap sizes of 4 nm, 14 nm, and 24 nm

To quantitatively characterize the gap-size dependence of the nanoplasmonic near-field generated by a BNA and evaluate the imaging performance of the plasmonic lithography with a BNA, we further investigate the enhanced near-field as a function of gap size via an electrodynamic model. According to the referenced theoretical near-field exposure model^22^, the patterning condition for the maximum pattern depth (*z*_max_) in plasmonic lithography is the dose modulation function (DMF) equal to the critical modulation transfer function (CMTF). The DMF is defined as *DMF* = (*D*_*max*_–*D*_*min*_)/(*D*_*max*_ + *D*_*min*_), where *D*_*max*_ and *D*_*min*_ represent the maximum and minimum exposure dose, respectively. The CMTF is defined as${{{\mathrm{CMTF}}}} = \left( {{{{\mathrm{e}}}}^{1/\gamma } - 1} \right)/\left( {{{{\mathrm{e}}}}^{1/\gamma } + 1} \right)$, where γ is the PR contrast and $\gamma = {{{\mathrm{ln}}}}\left( {{{{\mathrm{D}}}}_{{{\mathrm{c}}}}/{{{\mathrm{D}}}}_{{{{\mathrm{th}}}}}} \right)^{ - 1}$, *D*_c_ and *D*_th_ are the clearing dose and threshold dose of the PR, respectively. Hence, the maximum pattern depth (*z*_max_) generated by a plasmonic BNA can be solved as ${{{\mathrm{z}}}}_{{{{\mathrm{max}}}}} \cong \frac{a}{{{{\mathrm{b}}}}}\left[ {\left( {\frac{{1 + {{{\mathrm{DMF}}}}}}{{1 - {{{\mathrm{DMF}}}}}}} \right)^{ - {{{\mathrm{b}}}}} - 1} \right] \cong \frac{a}{{{{\mathrm{b}}}}}\left[ {\left( {\frac{{{{{\mathrm{D}}}}_{{{{\mathrm{max}}}}}}}{{{{{\mathrm{D}}}}_{{{{\mathrm{min}}}}}}}} \right)^{ - {{{\mathrm{b}}}}} - 1} \right]$, where *a* is the decay constant at *z* *=* *0*, and *b* is a dimensionless parameter, the constants *a* and *b* depend on the gap size of the plasmonic BNA. To record a pattern with a certain depth in the PR layer, the exposure dose is evaluated as *D*_*i*_*(g)* *=* *I*_*i*_*(g)t*_*ex*_, where *I*_*i*_*(g)* is the intensity at exposure depth *z* *=* *0* with a ridge gap size of *g*, and *t*_*ex*_ is the exposure time. It has been proven theoretically that the patterning condition in near-field lithography must meet the following requirements: *D*_*i*_*(g)* *=* *D*_max_*(g)*, and *D(z,g)* *≧* *D*_*th*_, where $D\left( {z,g} \right) = I_i\left( g \right)\left( {1 + \frac{b}{a}z} \right)^{ - 1/b}t_{ex}e^{ - \alpha z}$ is the exposure dose at exposure depth *z* with a ridge gap size of g, and α is the absorption coefficient of the used PR. Then, the maximum pattern depth (*z*_max_) can be redefined as

1 $${{{\mathrm{z}}}}_{{{{\mathrm{max}}}}} \cong \frac{a}{{{{\mathrm{b}}}}}\left[ {\left( {\frac{{{{{\mathrm{I}}}}_{{{\mathrm{i}}}}\left( {{{\mathrm{g}}}} \right)}}{{{{{\mathrm{I}}}}_0}} \cdot \frac{{{{{\mathrm{I}}}}_0{{{\mathrm{t}}}}_{{{{\mathrm{ex}}}}}}}{{{{{\mathrm{D}}}}_{{{{\mathrm{th}}}}}}}} \right)^{ - {{{\mathrm{b}}}}} - 1} \right]$$

where *I*_*0*_ is the magnitude of the incident electric-field intensity. In this study, a field enhancement factor (*F*) is specified to accurately predict the strength of the near-field enhancement accurately at the extremity of a plasmonic BNA^44,45^,

2 $$F{{{\mathrm{ = }}}}\left| {\frac{{I_i\left( g \right)}}{{I_0}}} \right| = \left| {\frac{{E_i\left( g \right)}}{{E_0}}} \right|^2$$

where *E*_*i*_*(g)* and *E*_*0*_ are the magnitudes of the enhanced electric-field in the PR layer and incident electric field, respectively. In previous work, a theoretical model with considering the finite-size effects was proposed to calculate the plasmonic near-field excited by a metal nanoparticle or a nanoridge aperture, and the field intensity enhancement can be calculated as a function of size *g*, *I*_*i*_*(g)* *=* *I*_*0*_*|1+ξ’|*^2^,^46^. Thus,

3 $$F = \left| {1 + \xi \prime } \right|^2$$

4 $$\xi \prime = \frac{\xi }{{1 - \left( {kg} \right)^2\xi - i\frac{2}{3}\left( {kg} \right)^3\xi }}$$

where *ξ* *=* *(ε–ε*_*m*_*)/(ε* *+* *2ε*_*m*_*), k* *=* *w/c* is the wavevector of the incident light, *ε*_*m*_ is the medium dielectric constant, and *ε* is the dielectric function of the metal. The dielectric function of the metal via the electron collision rate$\gamma = \gamma _{{{{\mathrm{bulk}}}}} + {{{\mathrm{Av}}}}_{{{\mathrm{f}}}}/{{{\mathrm{g}}}}$ can be expressed by the well-known Drude model $\varepsilon \left( \omega \right) = \varepsilon _\infty - \omega _{{{\mathrm{p}}}}^2/\left( {\omega ^2 + {{{\mathrm{i}}}}\omega \gamma } \right)$, where $\gamma _{{{{\mathrm{bulk}}}}}$ is the interband damping rate, *v*_f_ is the Fermi velocity of electrons, A is a phenomenological constant, and *ε*_*∞*_ and *ω*_*p*_ are the high-frequency dielectric constant and bulk plasma frequency of the metal, respectively (more details of the derivation of Eqs. (3) and (4) are shown in Supplementary Section S1). Figure 3a displays the enhancement factor of the electric field as a function of the nanoridge gap sizes from 4 nm to 40 nm. The results of the theoretical analysis on the field enhancement factor agree well with the simulations based on the transmission efficiency calculation. The transmission amplitude |T| as a function of gap size is calculated by the commercial software Lumerical FDTD, due to the advantages of Lumerical FDTD in the calculation of three-dimensional field distribution. Notably, the near-field enhancement factor exhibits a volcano trend with the nanoridge gap size, and the excited near-field intensity increases with the nanoridge gap size, reaching a maximum at a size of 20 nm, followed by a decrease at larger sizes. The physical reasons for the volcano-type size dependence of plasmonic near-field enhancement can be well explained from two aspects: first, the smaller nanoridge gaps show weak fields due to the surface damping of electrons; in contrast, the larger nanoridge gaps have reduced field enhancements due to the strong radiative scattering, and the other is the trade-off between the mode volume and the transmission efficiency. According to the proposed electrodynamic model (Supplementary Section S1), there are three size-dependent effects play critical roles in the enhancement of the plasmonic near-field: surface scattering, dynamic depolarization, and radiative decay. At small sizes, the near-field enhancement suffers from the additional surface damping due to the scattering of electrons with the nano-ridge gap surface. On the other hand, larger nano-ridge gaps have reduced field enhancements due to an increasing rate of radiative scattering, and dynamic depolarization also can cause damping fields at large nano-ridge gaps. Thereby, the interplay of all these effects gives rise to a volcano-type size dependence of near-field enhancement.

The cross-sectional *|E*_*xy*_*| and |E*_*xz*_*|* profiles of the excited plasmonic near-field generated by a BNA are shown in the right top and lower of Fig. 3a, respectively. In the *xy*-plane, the surface current arises from one ridge, flows around the BNA, and ends on the opposite ridge, and thereby deposits the electric charges with opposite signs on the two ridges. As a result, two counter-propagating antisymmetric metal-insulator-metal (MIM; Al-air-Al) SPP mode is polarized along the *x*-direction, and the excited SPP mode provides longer cutoff wavelengths which can yield larger transmission in the *z*-direction. Due to the excitation of the SPP mode, the excited EM field is enhanced and localized at the gap around and the gap size decides the field confinement. Nevertheless, the rapid divergence of the light scattered from the BNA results in a conflict between the antisymmetric MIM SPP mode volume and the transmission efficiency^29^. As the nanoridge gap size is reduced, the electric field tends to be concentrated in the vicinity of the central gap, and the corresponding MIM SPP mode volume decreases; conversely, the transmission efficiency is relatively enhanced, which ultimately results in a gap-size-dependent plasmonic field-coupling effect in plasmonic lithography. More importantly, the size dependence of the plasmonic near-field implies that one of the most significant contributors to the near-field OPE in plasmonic nanopatterning is the complicated decay characteristics of the evanescent field.

For a near-field imaging system, it is believed that the contributions of evanescent waves dominate the imaging performance, i.e., the higher evanescent wave (high-*k* information) transmissions are, the better the imaging performance that can be obtained at a certain high-*k* mode^25,40,47^. Thus, to further investigate the effect of the size dependence of plasmonic near-field enhancement on the imaging performance, the transmitted transverse (*E*_*x*_) and longitudinal (*E*_*z*_) electric field components are calculated with various nanoridge gap sizes, in which the ratio between the *|E*_*x*_*|* and *|E*_*z*_*|* determines the imaging fidelity. For a TM polarized incident wave propagating in the *xz*-plane, the total electric field intensity of the generated image can be calculated by *|E*_*x*_*|*^2^*+|E*_*z*_*|*^*2*^, i.e., $E_x(z)\sim k_x \cdot \partial H_y/\partial z$, and $E_z(z)\sim k_z \cdot \partial H_y/\partial z$, *k*_*x*_ and *k*_*z*_ are the wavevectors with $k_x^2 + k_z^2 = \varepsilon _{PR}k_0^2$. Due to the possible phase delay between the *|E*_*x*_*|*^*2*^ and *|E*_*z*_*|*^*2*^ for high-*k* evanescent waves^41^, if the ratio between them goes to 1, a blurred image will be obtained on the PR surface; therefore, a high ratio of *|E*_*x*_*|*^*2*^ */|E*_*z*_*|*^*2*^ is desired to record an image with good fidelity^28,40,48,49^. As shown in Fig. 3b, the transmission amplitudes of *|E*_*x*_*|* and *|E*_*z*_*|* are effectively modulated by the geometric characteristics of the plasmonic BNA. The modulation leads the component *|E*_*x*_*|* to be dominant in the image plane, and the negative imaging contribution of component *|E*_*z*_*|* is depressed as the gap-size-dependent plasmonic field-coupling effect. This field modulation effect by the plasmonic BNA with different nanoridge gap sizes helps to improve the image resolution and fidelity.

## Analytical estimation of near-field OPE for highly uniform patterns in plasmonic lithography

### Physical understanding of the near-field OPE

As one kind of maskless lithography, plasmonic lithography presents significant advantages for fast prototyping and high-cost efficiency without a physical mask, and has broad prospects for applications in the nanomanufacturing field with complicated and diverse pattern shapes. However, when a plasmonic lithography system generates a target pattern with arbitrary shapes, the physical mask is replaced by an analog target pattern image, which is converted into a pixel-based digital binary map for controlling the *ON/OFF* of the optical source. Therefore, the generated pattern is the result of the convolution of the PSF and the binary exposure map *B*_*n*_*(x, y)*. The exposure dose *D*_*arb*_*(x, y)* can then be given by:

5 $$D_{arb}\left( {x,y} \right) = \mathop {\sum}\limits_{i = 1,j = 1}^{N_{\exp }} {B_n\left( {x_i,y_j} \right) \times I_{psf}\left( {x - x_i,y - y_j} \right)} \times {{{\mathrm{t}}}}_n$$

where *N*_*exp*_ is the total number of the exposed pixels, (*x*_*i*_, *y*_*j*_) denotes the exposed pixel locations, *I*_*psf*_*(x, y)* is the intensity distribution of the PSF generated by the plasmonic BNA, and *t*_*n*_ represents the exposure time of one pixel. Equation (5) indicates that the exposure dose of an arbitrary pattern at a given location is not only dependent on the dose delivered at that location but also depends on the dose delivered at nearby locations. Therefore, precise control of the exposure dose is critical to achieving high resolution and quality in plasmonic lithography.

**Fig. 4:**
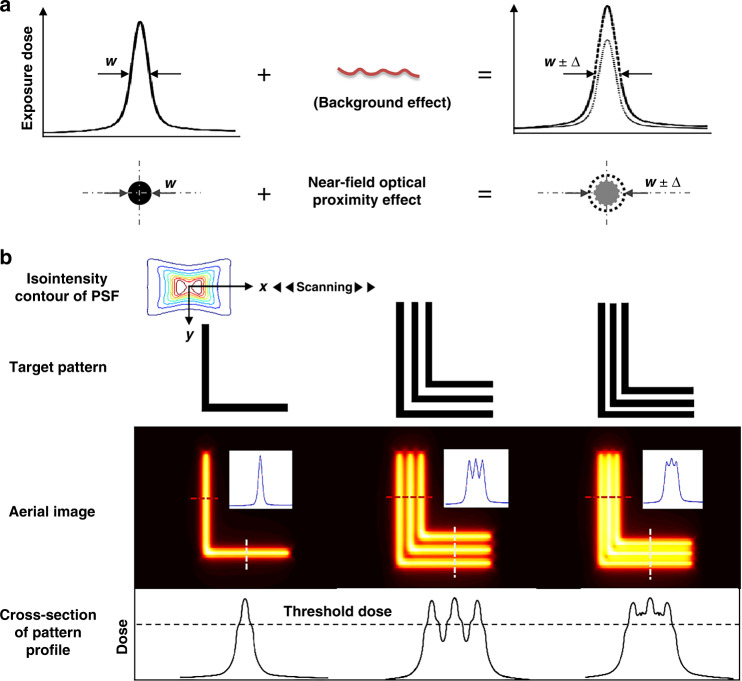
Physical understanding of the near-field optical proximity effects in plasmonic lithography. **a** An illustration of the effect of the background effect (i.e., optical proximity effect) on the pattern width (*w*). Background exposure dose arising from the spatial extent of the PSF contributes to the total exposure dose delivered at nearby exposed pixels, yielding a variation in the pattern width (*w* ± Δ). **b** Near-field OPE can result in an inability to record high dense features if they are sufficiently close-packed. Because the spatial distribution of the PSF is complex and asymmetric, the image distortions caused by the near-field OPE along the horizontal and vertical directions are also different

It is widely known that all forms of optical lithography exhibit distortion in the final image quality due to light diffraction and the finite aperture of the optics. In scanning near-field lithographic systems, this effect arises from the spatial extent of the PSF, i.e., the PSF can extend in space and contribute to the total exposure dose delivered at nearby locations. Thus, due to the spatial spreading of the PSF, the exposure dose from neighboring regions of the pattern can “spill over” and give rise to the near-field OPE. In light of this, the near-field OPE can be a significant source of error in the patterning results. The origin of the near-field OPE in plasmonic lithography is illustrated in Fig. 4. Figure 4a illustrates that the background effect refers to the near-field OPE, which is the spatial extent of the PSF contributing to the total exposure dose delivered at nearby exposed pixels. In turn, the contribution of the near-field OPE can result in adding a gain component to the PSF. Since the pattern profile is determined by a high-energy volume over the threshold dose of PR, under the condition of a certain threshold dose of PR, the addition determined by the near-field OPE further leads to variations in pattern width. In Fig. 4b, the simulation results show how the near-field OPE can make it difficult to obtain a high-dense array pattern with good fidelity. Because the complex and asymmetric field structure of the PSF, two sharp intensity peaks at the nanoridge gap edges and complicated decay characteristics with respect to variations in the planar plane lead to the effects of the near-field OPE on image distortions presenting asymmetrically and drastically changing in the *x*- and *y*-directions. Significantly, if the characteristics of the PSF can be quantitatively analyzed, especially its decaying features (each decaying feature may can be associated with a distinct spatial frequency), and given that recording in exposure results from the convolution of the target pattern to be exposed with the PSF, the required exposure dose of each pixel can be accurately calculated to achieve the desired final exposure dose at each exposed pixel, which is regarded as the pixel-based dose modulation method. However, the precise control of feature size modulation suffers from the loss of spatial frequency information, due to the near-field decays rapidly, i.e., near-exponentially with increasing distance *z* from the aperture exit. Hence, the quantitative relationships between the decaying feature and the spatial frequency in near-field patterning systems have not yet been intensively studied.

### Effect of the decaying feature on the near-field OPE

**Fig. 5:**
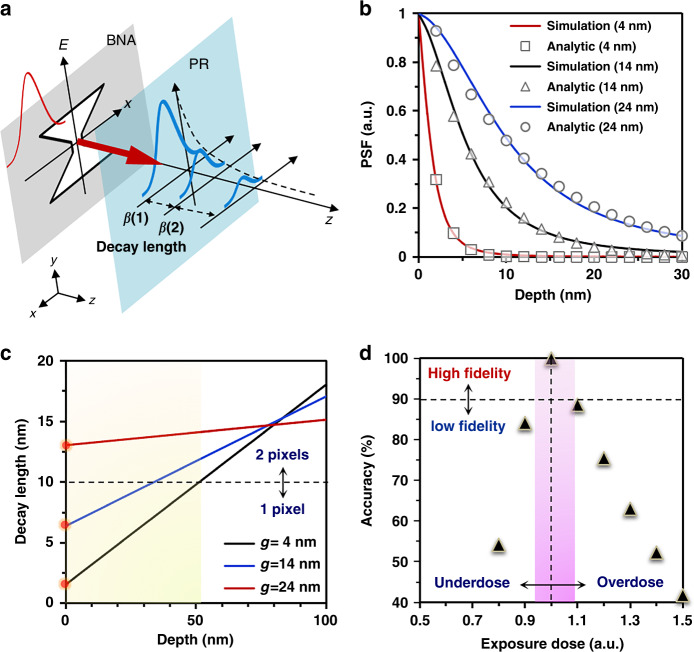
The rapidly decaying features of the evanescent field in the PR layer and the effect of the decaying features on the pattern quality. **a** Schematic diagram showing the decay characteristics of the evanescent waves. The decay length is not a constant but varies with the exposure depth. **b** PSFs in the PR layer generated by plasmonic BNA with different nanoridge gap sizes. **c** Estimation of the decay lengths determined by different nanoridge gap sizes of 4 nm, 14 nm, and 24 nm and their different effects on the exposed pixel. **d** Pattern accuracy as a function of the exposure dose

Generally, pattern quality in optical lithography suffers from the missing of the high-*k* information (i.e., evanescent wave components) while passing through the exposure systems. However, the loss of high-*k* information in plasmonic lithography is associated with the rapidly decaying feature of the evanescent wave amplitudes with increasing exposure distance and the propagating mode of the QSWs in the PR layer. Then, according to the near-field lithography model, the spatial frequencies associated with the propagating and evanescent field components in the PR layer (*k*_*z*_*(z)*) can be represented by the following formula^22,41,50,51^:

6 $$k_z\left( z \right) = {\int_0^z {\frac{{dz}}{{\beta \left( z \right)}}}}$$

where *β(z)* is the decay length and can be approximated by a linear function of *β(z)* *=* *a+bz*, and the constants *a* and *b* are dependent on the geometry of the evanescent field and can be determined by fitting the peak intensity of the electric field distribution along the *z*-direction in the PR layer. As described in Fig. 5a, the decay length of the near-field intensity in the PR layer is not a constant, and changes with the exposure depth (Supplementary Section S2). Meanwhile, as the decay length increases with the exposure depth, the intensity of the spatial frequency decreases. This result indicates that the attenuation of the high-*k* information carried by the evanescent field is more rapid than that of the low-*k* (i.e., propagating wave components), which is the main optical contributor to the near-field OPE, thus yielding pattern distortion in the exposure process. Therefore, to improve the pattern quality, it is important to quantitatively analyze the evanescent-field-induced high-*k* loss on the basis of the quantitative characterization of the decay length of the evanescent field.

In particular, the gap size of a plasmonic BNA plays a very significant role in determining the spatial distribution of the PSF in the PR layer. The PSFs generated by different nanoridge gap sizes are further investigated to verify the decay characteristics of the evanescent field. As shown in Fig. 5b, the fitting results based on the simple analytic decay function *β(z)* agree well with the simulation results. The parameters (*a, b*) of the decay length *β(z)* for different nanoridge gap sizes of 4, 14, and 24 nm are fitted as (1.549 nm, 0.165), (6.377 nm, 0.107), and (13.043 nm, 0.021) from the PSF curves, respectively. Moreover, to gain physical insight into the effect of the evanescent field-induced loss of high-*k* information on the pattern fidelity, the effect of the decay length on the exposed pixel number is quantitatively estimated. The calculated decay length curves for several plasmonic BNAs with nanoridge gap sizes of 4, 14, and 24 nm, extracted from the corresponding PSFs, are plotted in Fig. 5c. It can be observed that the decay length at *z* *=* *0* increases with the gap size; however, it decreases as the gap size increases at a certain exposure depth (i.e., far-field region). The main reason for this is that the rapid loss of the high-*k* information along the exposure depth can significantly weaken the evanescent field components and the propagating field components are gradually dominant in the far-field exposure region. In fact, the area affected by the near-field OPE can be determined by the decay length of the evanescent field. To be more specific, if the pixel size of the binary image is set as 10 nm, the affected areas by the near-field OPE are estimated to be 1 pixel and two pixels for different plasmonic BNAs with nanoridge gap sizes of 4, 14, and 24 nm, respectively. The determination of the affected area is critical to improve the image distortion problem, because it would significantly reduce the optimization time.

Previously, it was demonstrated that the pattern profile exposed by the plasmonic lithography system strongly depends on the required exposure dose; thus, precisely and spatially modulating the feature size with the optimal exposure dose is a key factor for a successful exposure. However, due to the diversity and complexity of the pattern shapes and the narrow exposure latitude (EL) of the near-field lithographic system, it is difficult to precisely control the optimal dose in the exposure process. In this study, with the aim of achieving precise control of the required exposure dose, the pattern accuracy at the nominal feature with ±10% CD variation is characterized by measuring the CD in the PR layer as a function of exposure dose, which can be formulated as

7 $$Accuracy = \left( {1 - |\frac{{\mathop {\sum}\limits_{i,\,j} {D_n\left( {x_i,y_j} \right) - B_n\left( {x_i,y_j} \right)} }}{{\mathop {\sum}\limits_{i,\,j} {B_n\left( {x_i,y_j} \right)} }}|} \right) \times 100\%$$

where *D*_*n*_(*x*_*i*_, *y*_*j*_) and *B*_*n*_(*x*_*i*_, *y*_*j*_) denote the exposed pattern map and the original binary pattern map, respectively. As shown in Fig. 5d, a small variation in the exposure dose can lead to a relatively large change in the pattern accuracy. Therefore, to overcome the near-field OPE in plasmonic lithography, the precise manipulation of features for nanopatterning with considering the decaying feature of the near-field is the basis to ensure the quality of the exposure profile. Furthermore, pattern distortion caused by the decay characteristic of the near-field affects its process of high accuracy. This has some limitations in its practical application.

## Results and discussion

### Patterning strategy for higher pattern quality in plasmonic lithography

**Fig. 6:**
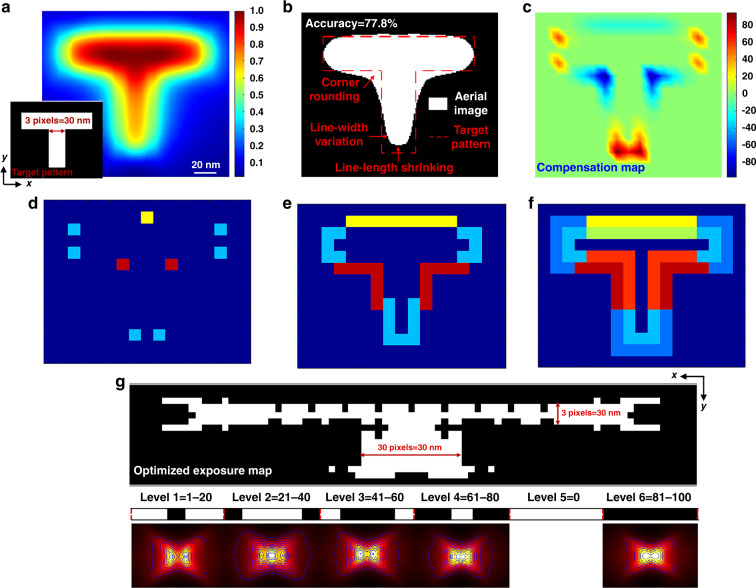
The pattern distortion of near-field OPE in plasmonic lithography and near-field OPC for high pattern quality. **a** Aerial image of a ‘T’ pattern generated by plasmonic lithography. Inset shows the original exposure map of the ‘T’ pattern. **b** Simulated profile of a ‘T’ pattern on the PR. **c** Compensation map for the ‘T’ pattern. **d**–**f**, illustrate a hybrid-model-based patterning strategy that follows the ‘point-line-surface’ process. **d** The initial regions assigned to the feature error points, i.e., corner rounding (red), line-width variation (yellow), and line-length shrinking (blue). **e** The initial regions assigned to the feature error lines, i.e. corner rounding (red), line-width variation (yellow), and line-length shrinking (blue). **f** The initial regions assigned to the feature error map, i.e. corner rounding (red), line-width variation (yellow), and line-length shrinking (blue). **g** The optimized exposure map with dose leveling (*N* = 6), the corresponding aerial images, and isointensity contours of the PSFs for dose leveling are also shown (bottom) (Supplementary Section S3)

Recording patterns at the nanoscale with low distortion is essential for plasmonic lithography; however, due to the near-field OPE, it becomes difficult for current plasmonic lithography to continue to reduce the node size with high pattern fidelity. The optical proximity effect correction (OPC) method can effectively solve this problem by adjusting the exposure dose delivered to each pixel to the appropriate level combined with shape modulation for the original exposure map of the target pattern image. As described in the previous section, the decaying features of the evanescent field lead to rapid high-*k* information loss while passing through the PR layer, thus yielding distortions of the final exposure patterns, for instance, corner rounding, line-width variation, and line-length shrinking. The simulation results of the aerial image and the simulated final exposure profile of a ‘T’ letter with the minimum line-width at three pixels are shown in Fig. 6a, b, respectively. The aerial image represents the spatial distribution of the optical intensity on the PR layer, which corresponds to the exposure condition of the PR. An accurate near-field lithography model which has demonstrated that the PR profile was properly matched with the calculated intensity of the isointensity contour by comparing the maximum pattern depth and width, is employed to obtain the final pattern profile^52^. Considering the decaying feature in the PR layer and the used PR parameters, such as the PR contrast (γ), threshold dose (*D*_*th*_), clearing dose (*D*_*cl*_), and the developing process, the analytic solution for the development profile *S*_*D*_(*x, y;z*) can be obtained as

8 $$S_D\left( {D_{ex}\left( {x,y;z} \right)} \right) = \left\{ {\begin{array}{*{20}{l}} {0,} \hfill & {{{{\mathrm{for}}\, {\mathrm{D}}}}_{{{{\mathrm{ex}}}}} \le D_{th},} \hfill \\ {\beta \left( z \right)\ln \left( {D_{ex}/D_{th}} \right)\left[ {1 - \exp \left( { - \gamma T_0/\beta \left( z \right)} \right)} \right],} \hfill & {{{{\mathrm{for}}\,{\mathrm{ D}}}}_{{{{\mathrm{th}}}}} \le D_{ex} \le {{{\mathrm{D}}}}_{{{{\mathrm{cl}}}}},} \hfill \\ {\beta \left( z \right)\ln \left( {D_{ex}/D_{th}} \right) - \beta \left( z \right)\left[ {\left( {D_{ex}/D_{th}} \right)^\gamma /e\gamma } \right]\exp \left( { - \gamma T_0/\beta \left( z \right)} \right),} \hfill & {{{{\mathrm{for}}\,{\mathrm{ D}}}}_{{{{\mathrm{cl}}}}} \le D_{ex},and} \hfill \\ {T_0,} \hfill & {{{{\mathrm{for}}\,{\mathrm{ D}}}}_{{{{\mathrm{cl}}}}}e^{T_0/\beta \left( z \right)} \le D_{ex}{{{\mathrm{.}}}}} \hfill \end{array}} \right.$$

where the *D*_*ex*_*(x,y;z)* is the exposure dose in the transverse direction *(x,y)* and decaying rapidly in the pattern depth (*z*) in the PR layer, *T*_*0*_ is the PR thickness, and *e* is Euler’s number. Given the exposure dose distribution at the plane *D*_*ex*_*(x,y;z)*, the PR removal thickness *S*_*D*_ as a function of position *(x,y;z)* can then be evaluated. It is obvious that the simulated pattern profile of the ‘T’ letter is different from the target image. Under the optimized exposure condition, the achievable accuracy is only approximately 77.8%, which makes it difficult to satisfy the requirement of high pattern fidelity (i.e., accuracy >90%). Figure 6c displays the exposure map, which needs to compensate for the shape errors between the target pattern and the final exposure pattern. The blue areas and red areas represent overdoses and underdoses, respectively. On the basis of the exposure map, the near-field OPE in plasmonic lithography can, in general, be corrected by pre-compensating the exposure map before patterning. Herein, we present a hybrid-model-based patterning strategy for exposure map design modulation and pattern fidelity optimization with an OPC concept in plasmonic lithography. A point-line-surface process (as shown in Fig. 6d–f) is first followed to define the regulatory regions based on the compensation map (Fig. 6c). As shown in Fig. 6d, the observation points of the three kinds of typical feature errors are marked by different colors. We first assign an initial region for each feature error observation point, and each feature error is iteratively extended from a single point to a line around it. In each iteration, all observation points are simultaneously extended by one pixel along the boundaries to both sides. Figure 6d shows that the extension terminates when all of the needed compensation regions are covered by the line regions. The initial regions of all observation points are finally extended to cover the affected area of decaying features and the decomposition map of feature errors is shown in Fig. 6e. Then a gradient-based algorithm is used to optimize the exposure map based on the cost function (*PE*_*c*_)^53^.

9 $$PE_c = \gamma _IPE_I + \gamma _RPE_R$$

10 $$PE_I = \left\| {I_t\left( {x,y} \right) - I_i\left( {x,y} \right)} \right\|^2$$

11 $$PE_R = \left\| {D\left( {I_t\left( {x,y} \right)} \right) - D\left( {I_i\left( {x,y} \right)} \right)} \right\|^2$$

where *PE*_*I*_ *and PE*_*R*_ represent the costs for the aerial image and final pattern profile, respectively. The norm $||\cdot||^2$ denotes the square of Euclidean distance which denotes the inner product of the same vector, and γ_I_ and γ_R_ are the weighting factors. An exposure map $\hat B$ optimization can thus be formulated as

12 $${{\hat {B}}} = \mathop {{{{{\mathrm{arg}}}}\,{{{\mathrm{min}}}}}}\limits_{B_n(x,y)} PE(B_n(x,y))$$

Generally, for a large optimization problem, the computation time is an important consideration, because all the pixels on the exposure map are basically modified. However, the proposed hybrid-model-based patterning strategy is faster than other gradient-based algorithms. The major reason is that the adjusted exposure map features are directly obtained by calculating the affected area caused by the evanescent-field-induced decay loss, which further shortens the run-time of the optimization process. Figure 6f displays the optimized exposure map with dose leveling (*N* = 6). Simulation results of the varied PSFs demonstrate that assist dose leveling can effectively relax the shape complexity of the near-field distribution and enable the exposure dose of each pixel to be controlled more precisely and flexibly.

### Patterning results with the near-field OPC-assisted patterning process

**Fig. 7:**
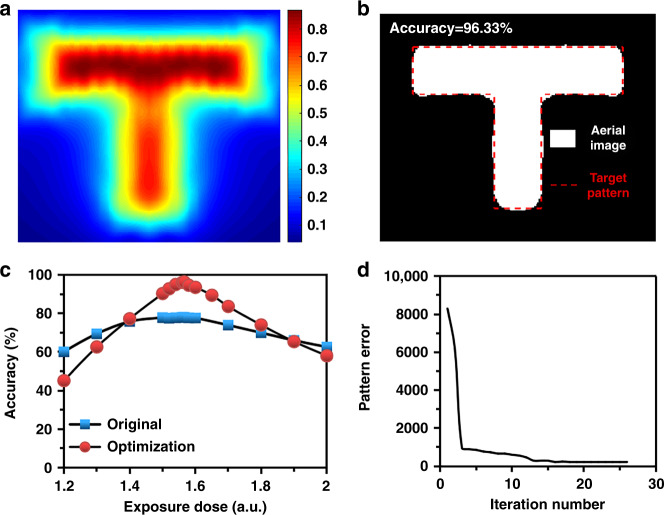
Simulated results using the near-field OPC patterning strategy. **a** Aerial image and **b** Simulated final exposure pattern of a ‘T’ pattern with OPC by dose leveling. **c** Comparison of the calculated accuracy for the original exposure map and the optimized exposure map. **d** Convergence curve of pattern error based on the ‘T’ pattern

The T-shaped aerial image and the simulated final pattern shape are calculated with OPC by dose leveling as described in Fig. 7a, b, respectively. By performing the OPC with a dose leveling structure, the achievable accuracy of the ‘T’ pattern can be enhanced to over 96%, which basically verifies the efficiency of the proposed hybrid-model-based patterning method. The entire pattern fidelity is effectively improved, including the corners, edges, and shortening effects. To precisely verify the effectiveness of the near-field OPC method, we further calculate the accuracy as a function of exposure dose for the original exposure map and the optimized exposure map of the ‘T’ pattern. As plotted in Fig. 7c, the accuracy curve for the original exposure map is broader than that for the optimized exposure map. This can be explained by the fact that without a modified exposure pattern, the generated aerial image is a nonlinear low-pass filtered version of the desired feature, due to the evanescent-field-induced high-*k* loss. However, the high-*k* loss induced by the rapidly decaying feature of the evanescent field can be pre-compensated by the near-field OPC with dose leveling. Figure 7d shows the convergence curve. The pattern errors can be effectively reduced by applying the OPC method with dose leveling, and the OPC method converges after approximately 15 iterations.

## Conclusion

In summary, we have explored the physical mechanism of near-field OPE in plasmonic lithography, and a hybrid-model-based OPC with dose leveling is proposed to enhance the pattern fidelity. Precise proximity effect correction requires accurate exposure control. By evaluating the PSF generated by a plasmonic BNA, the complicated decay characteristic and the asymmetry of the electric-field distribution of the near-field beam spot can be quantitatively studied. Theoretical and simulation results indicate that the rapid loss of high-*k* information induced by the evanescent field is one of the main optical factors influencing the final pattern accuracy in plasmonic lithography. Using quantitative analysis of the decaying feature of the evanescent field, quantitative correction of exposure dose compensation is conducted, and after the patterning process, the nanoscale CD errors are significantly reduced. These results demonstrate new possibilities for dramatically improving the final pattern fidelity with arbitrary shapes at the nanoscale using plasmonic lithography.

## Supplementary information

Supplementary Information
