# Supplementary material for: Enhancement of pattern quality in maskless plasmonic lithography via spatial loss modulation
Source: Microsyst Nanoeng. 2023 Mar 30;9:40. doi: 10.1038/s41378-023-00512-4 (PMC10060221; doi:10.1038/s41378-023-00512-4)
Supplement: Supplementary file 1 — Supplementary Information [file 41378_2023_512_MOESM1_ESM.docx]

- Dandan Han, Sen Deng, Tianchun Ye*, and Yayi Wei*

**Supplementary information**

Enhancement of pattern quality in maskless plasmonic lithography via spatial loss modulation

**S1 Theoretical model of plasmonic near-field generated by a bowtie nanoaperture**

Bowtie-shaped nanoaperture (BNA) has been exploited as a plasmonic nanostructure for improving the field enhancement in the optical and photovoltaic applications. Extensive works have focused on the optimization of plasmonic near-field by interparticle coupling and geometric tuning of nanoparticles, but the effect of gap size on the transmission of the plasmonic near-field generated by a metallic BNA has received less attention.

The spatial distribution of the electric field generated by a plasmonic BNA can be approximately calculated by the photopolymerization volume^1-6^, i.e., Transmission ≈ Photopolymerization volume = [Plasmonic field area] × [Max. Z] = AFM measured [FWHM (x)] × [FWHM (y)] × [Max. Z] ≈ [～nm^3^], where [FWHM (x)] and [FWHM (y)] are the full width at half maximum (FWHM) in the x- and y- directions, and [Max. Z] is the maximum pattern depth, as shown in Fig. S1.


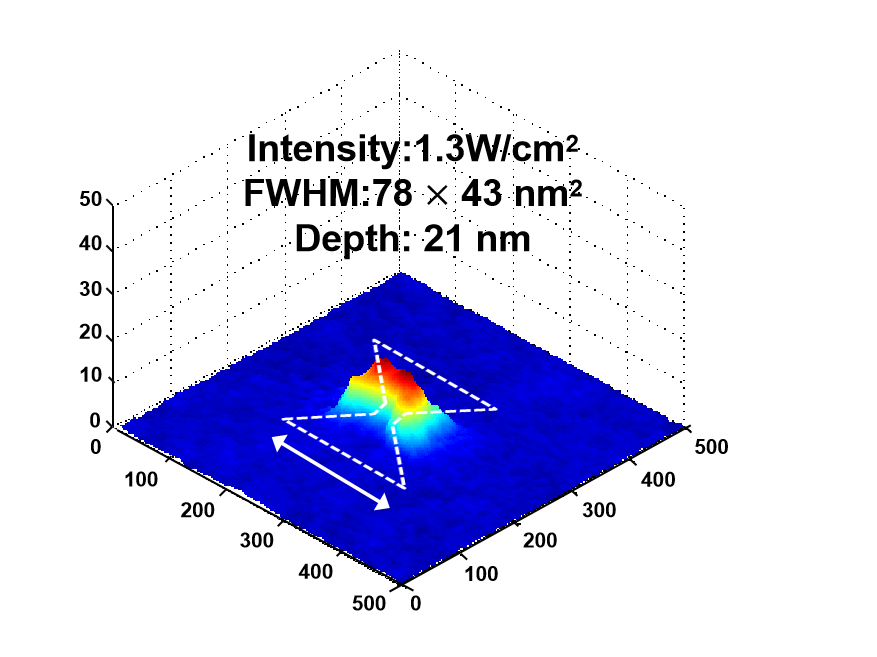


**Fig. S1.** A three-dimensional profile of developed pattern measured by AFM^4^.

According to the near-field exposure model, the maximum pattern depth (Z_max_) can be expressed as

$$\begin{matrix} Z_{\max}\cong\left(\frac{a}{b}\right)[(\frac{1+DMF}{1-DMF})^{-b}-1]\text{ (}∵DMF=\frac{D_{\max}-D_{\min}}{D_{\max}+D_{\min}}\text{ })\text{ } \\ \text{ =}\left(\frac{a}{b}\right)[(\frac{D_{\max}}{D_{\min}})^{-b}-1]\text{ (} ∵\text{ Near-field patterning condition: D}_{i}\text{(g)=D}_{\max}\text{(g); D(z,g)}\geq\text{D}_{th} \\ \text{ =}\left(\frac{a}{b}\right)\left[\left(\frac{I_{i}(g)\times t_{\exp}}{D_{th}}-1\right)^{-b}\right]\text{ } \therefore\text{ D}_{\text{max}}=I_{i}(\text{g})\times t_{\exp},D_{\min}=D_{th}) \\ \text{ }=\left(\frac{a}{b}\right)\left[\left(\frac{I_{i}(g)}{I_{0}}\frac{I_{0}\times t_{\exp}}{D_{th}}\right)^{-b}-1\right]\text{ } \\ \text{ } =\left(\frac{a}{b}\right)\left[\left(F\frac{I_{0}\times t_{\exp}}{D_{th}}\right)^{-b}-1\right]\text{ (1)} \end{matrix}$$

where a is the decay constant at *z=0*, b is a dimensionless parameter, the dose modulation function (DMF) is defined by the maximum exposure dose (D_max_) and minimum exposure dose (D_min_), the exposure dose is evaluated as *D_i_(g)=I_i_(g)t_ex_*, where *I_i_(g)* is the intensity at exposure depth *z=0* with a ridge gap size of *g*, *t_ex_* is the exposure time, *D(z,g)* is the exposure dose at the exposure depth *z*, *D_th_* are the threshold dose of the photoresist, and *I_0_* is the magnitude of the incident electric-field intensity*. F*, which refers to the field enhancement factor, and is specified to predict the strength of the plasmonic near-field^7^.

$$F\text{=}\left|\frac{I_{i}\left(g\right)}{I_{0}}\right|=\left|\frac{E_{i}\left(g\right)}{E_{0}}\right|^{2}\text{ (2)}$$

where *E_i_(g)* and *E_0_* are the magnitudes of enhanced electric-field in the PR layer and incident electric field, respectively. Because the plasmonic BNA consists of two open arms and a sharp ridge, when a polarized plane wave is illuminated on its entrance plane, localized surface plasmon polariton (SPP) mode around the BNA can be excited and form a new electromagnetic field. The accumulated charges oscillate with the excitation electromagnetic field and act as an electric dipole, thus it can be assumed as a two particle system^8^.

In general, since the plasmon oscillations excited by a metal nanoparticale can induce a nonradiative damping of the near-field, the excitation of plasmon resonances can be described by its dipolar polarizability *α*, and the excited near-field amplitude can be described as *E_nf_=E_0_|1-ξ|.* Thus, based on the quasi-static approximation, the *E_i_(g)* generated by a plasmonic BNA can be expressed as

$$\begin{matrix} E_{i}(R)=E_{0}+\frac{\mu'}{g^{3}} ; \mu=\alpha E_{0}, \mu'=\alpha'E_{0} \\ =E_{0}+\frac{\alpha'E_{0}}{g^{3}} \\ =E_{0}+\xi'E_{0} \\ =E_{0}(1+\xi') \\ \therefore\text{ }F=\left|1+\xi'\right|^{2}\text{ (3)} \end{matrix}$$

where α is the dipolar polarizability, µ is the dipole moment, $\mu^{'}$is the induced dipole moment, $\alpha^{'}$is the modified polarizability. Thus, in order to obtain $\xi^{'}$, we first need to derive the dipolar polarizability (α) of plasmonic BNA. Considering the electric field polarization of the impinging plane wave, the dipolar polarizability equation of the plasmonic BNA is as follows^9,10^:

$$\begin{matrix} \alpha=V\frac{\varepsilon-\varepsilon_{m}}{\varepsilon_{m}+L(\varepsilon-\varepsilon_{m})}=\frac{1}{3}d\left[a(2b+\text{g})-c\text{g}\right]\left(\frac{\varepsilon-\varepsilon_{m}}{\varepsilon_{m}+L(\varepsilon-\varepsilon_{m})}\right)\text{ (4)} \\ L=\frac{3}{\pi}\tan^{-1}\left\{\frac{a\cdot\text{d}}{\left(b+\frac{g}{2}\right)\sqrt{a^{2}+4\left(b+\frac{g}{2}\right)^{2}+\text{d}^{2}}}\right\}\text{ (5)} \end{matrix}$$

where V is the particle volume, ε is the dielectric function of the metal, ε_m_ is the medium dielectric constant, and L is the depolarization factor.


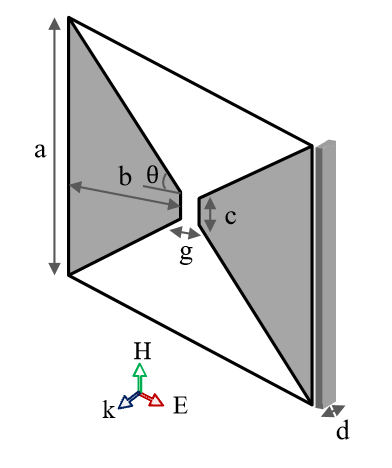


**Fig. S2.** Geometrical sketch of the plasmonic BNA.Geometrical parameters: $a=2btan\theta+c.$ d is the thickness of Al film.

More importantly, for the plasmonic near-field generated by a metallic nano-ridge structure, two additional size-dependent effects also play a role in the decay of the near-field. One is the radiative decay effect caused by the far-field radiation of light by the oscillating field. The rate of the radiative damping increase with the nano-ridge gap size as *(kg)^3^*, where *k=w/c* is the wavevector of the incident light. The other one is the dynamic depolarization effect exhibited by the finite-sized nano-ridge structure, resulting from the phase retardation of the exciting field with respect to the nano-ridge structure^6^. Hence, the additional radiation field induced by the dynamic depolarization and radiative decay E_rad_ can be expressed as:

$$E_{rad}=\left(\frac{k^{2}}{g}+i\frac{2}{3}k^{3}\right)\mu' \text{ (6)}$$

The plasmonic near-field is modified by the E_rad_, and the induced dipole moment µ'=α(E_0_+E_rad_). Thus, the modified polarizability α'=µ'/E_0_ is

$$\begin{matrix} \alpha'=\frac{\alpha}{1-\frac{k^{2}}{g}\alpha-i\frac{2}{3}k^{3}\alpha}\text{ (7)} \\ \xi'=\frac{\alpha'}{g^{3}}=\frac{\xi}{1-(k\text{g})^{2}\xi-i\frac{2}{3}(kg)^{3}\xi}\text{ (8)} \end{matrix}$$

where ξ=α/g^3^=(ε-ε_m_)/(ε+2ε_m_). Therefore, from the modified polarizability α', we can calculate the field enhancement factor F=|1+ξ'|^2^ generated by a plasmonic BNA as a function of gap size (g).

**S2 Decay properties of the propagating and non-propagating electric fields**

As shown in Fig. S3a, for a propagating electric field, the electric field strength can be propagated to far-field without decay. However, for a non-propagating electric field generated by an object illustrated in Fig. S3b, the electric field *E(x,y,z)* can be expressed as

$$E (x,y,z)= I(x,y,z)\times t = E_{0} e^{-\left|k_{z}\right|z}\text{ (9)}$$

According to the dispersion relation,

$$\begin{matrix} k^{2}=k_{x}^{2}+k_{y}^{2}+k_{z}^{2}=\frac{\omega^{2}}{c^{2}}\varepsilon\text{ (10)} \\ k_{z}=\sqrt{\frac{\omega^{2}}{c^{2}}\varepsilon-\left(k_{x}^{2}+k_{y}^{2}\right)}\text{ (11)} \end{matrix}$$

*k_z_* becomes imaginary, if (w^2^/c^2^)ε < (k^2^_x_+k^2^_y_). Thus, the electric field exponentially decays along z direction, i.e., I(z)=I_i_e^-2|kz|z^=I_i_e^-z/β^, the decay length *β* is constant and only one specific spatial frequency in the near-field. For a near-field generated by a plasmonic BNA, as shown in Fig. S3(c), the decay length β is not a constant, it is an average of the continuous spectrum of decay lengths (each of them being associated with the lateral wave vectors). Thus, the intensity distribution from the plasmonic BNA is not a simple exponential decay function, the decay length *β(z)* changing with the distance *z*, and can be assumed using a linear function of *β(z)=a+bz*.


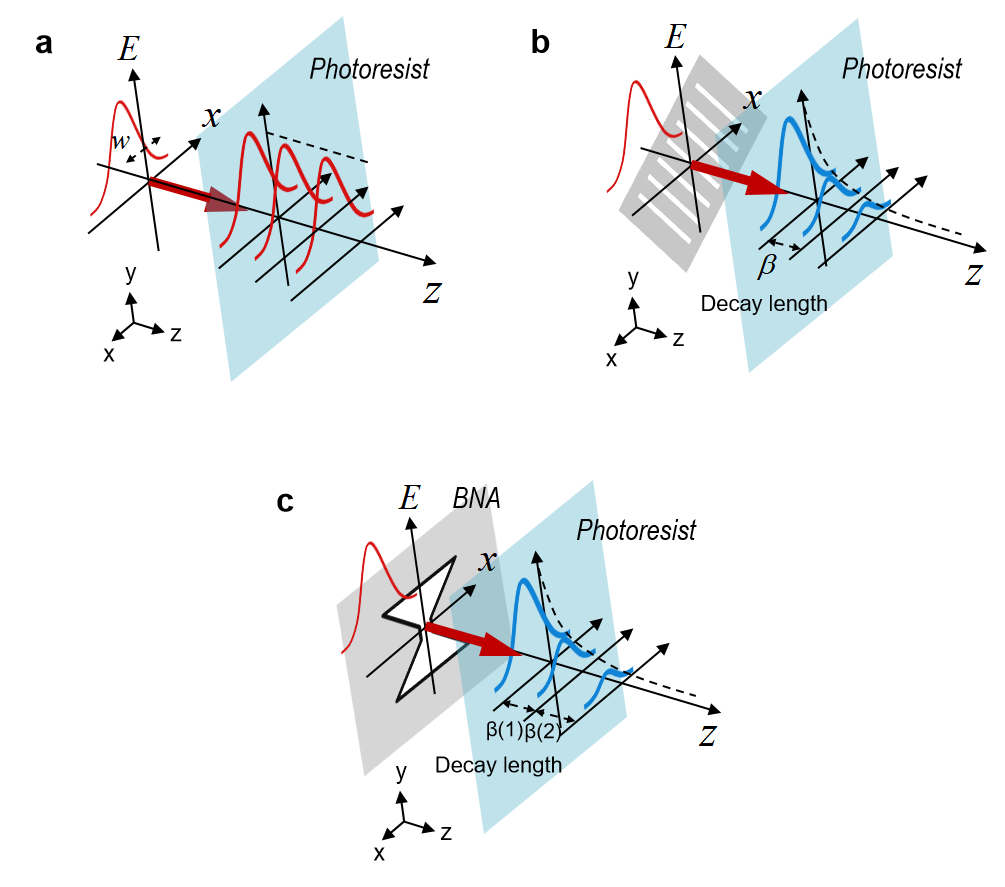
**Fig. S3.** **a** Schematic of the propagating electric field without field decay. **b** Schematic of the non-propagating electric field with a single decay length *β*. **c** Schematic of the non-propagating electric field with a various decay lengths *β(z)*.

**S3 Spatial modulation associated with sub-pixel in maskless plasmonic lithography**

In plasmonic grayscale lithography with a scanning BNA, continuous exposure is available along the scanning direction within a given line. During the exposure process, the input laser power is kept constant as well as focusing setting. Thus, the accumulated dose (*D*) along the scanning direction within the laser On-pixel region can be expressed as follows:

$$\begin{matrix} D(x,y,z,t)=\int_{0}^{t_{s}} I\left(x_{s}-vt,y,z\right)dt, \\ t_{s}=\frac{\Delta}{v}, \\ \Delta=\frac{P_{S}}{n_{s}}.\text{ (12)} \end{matrix}$$

where *I(x,y,z,t)* is the designed grayscale intensity distribution, *t* is the exposure time, *x_s_* is the patterning length along the scanning direction, *v* is the scanning speed, *t_s_* is determined by the scanning speed and the sub-pixel size $(\Delta)$, and $\Delta$ is determined by the pixel size (*P_s_*) and the step level (*n_s_*). During a scan within a pixel size, the maximum number of laser On-unit times depends on the *n_s_*. For instance, when *n_s_=5*, the maximum number of laser On-unit times is *5*, the leveling number *N=6*. As illustrated in Fig. S4, the dose control is based on the spatial modulation of laser pulse width. For accurate controlling the pattern feature size, discrete sub-pixel is used as an assist feature to deliver the proper dose to the adjacent region and not appear as developed pattern.

**
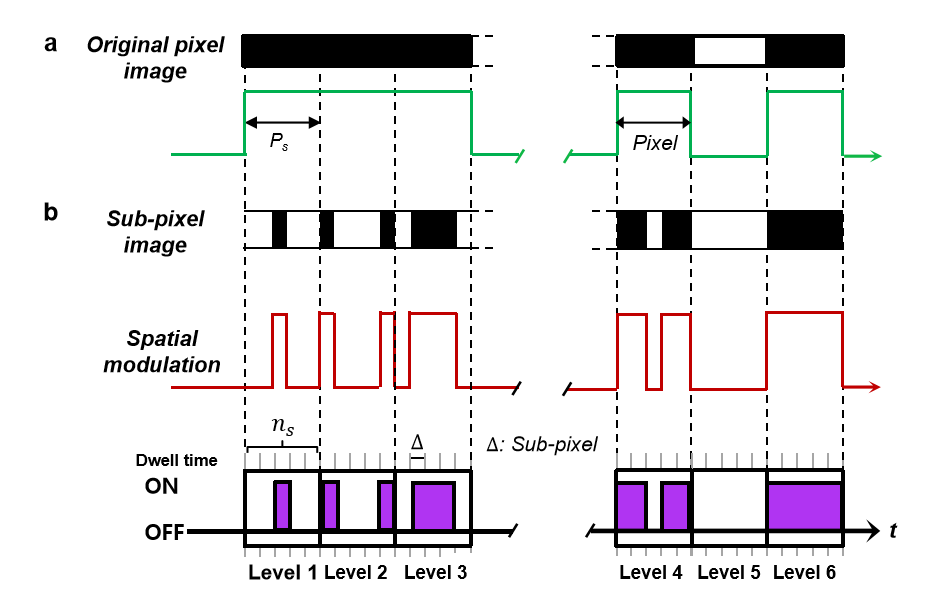
**

**Fig. S4.** **a** Schematic of spatial modulation of pattern feature size using a binary image without dose leveling. **b** Schematic of spatial modulation of pattern feature size with dose leveling.

**Reference**

1. Ecoffet, C., Espanet, A., and Lougnot, D. J. Photopolymerization by evanescent waves: a new method to obtain nanoparts. *Adv. Mater.* **10**, 412–414 (1998).
2. Amarie, D., Rawlinson, N. D., Schaich, W. L., Dragnea, B., and JacobsonIto, S. C. Three-dimensional mapping of the light intensity transmitted through nanoapertures. *Nano Lett.* **5**, 1227-1230 (2005).
3. Deeb, C., Bachelot, R., Plain, J., Baudrion, A., Jradi, S., Bouhelier, A., Soppera, O., Jain, P. K., Huang, L., Ecoffet, C., Balan, L., & Royer, P. Quantitative analysis of localized surface plasmons based on molecular probing. *ACS Nano* **4**, 4579–4586 (2010).
4. Kim,Y., Jung, H., Kim, S., Jang, J., Lee, J. Y., & Hahn, J. W. Accurate near-field lithography modeling and quantitative mapping of the near-field distribution of a plasmonic nanoaperture in a metal. *Opt. Express* **19**, 19296-19309 (2011).
5. Kim, S., Jung, H., Kim, Y., Jang, J., & Hahn, J. W. Resolution limit in plasmonic lithography for practical applications beyond 2x-nm half pitch. *Adv. Mater.* **24**, OP337–OP344 (2012).
6. Deeb, C., Zhou, X., Plain, J., Wiederrecht, G. P., & Bachelot, R. Size dependence of the plasmonic near-field measured via single nanoparticle photoimaging. *J. Phys. Chem. C* **117**, 10669-10676 (2013).
7. Yin, H., Zhang, J., Wang, X., Cui, J., Wang, W., & Mei, X. Recent progress in near-field tip enhancement: principles and applications. *Phys. Status Solidi RRL* **16**, 2100456 (2022).
8. Park, C., Jung, H., & Hahn, J. W. Characterization of three-dimensional field distribution of bowtie aperture using quasi-spherical waves and surface plasmon polaritons. *Sci. Rep.* **7**, 45352 (2017).
9. Jain, P. K., and El-Sayed, M. A. Noble metal nanoparticle pairs: effect of medium for enhanced nanosensing. *Nano Lett.* **8**, 4347–4352 (2008).
10. Iovine, R., Spada, L. L., Vegni, L. Modified bow-tie nanoparticles operating in the visible and near infrared frequency regime. *ANP* **2**, 21-27 (2013).
